# Establishing reference interval for thyroid-stimulating hormone in children below two-year ages in Pakistani population

**DOI:** 10.1016/j.amsu.2021.102601

**Published:** 2021-07-31

**Authors:** Siraj Muneer, Imran Siddiqui, Hafsa Majid, Lena Jafri, Khadija Nuzhat Humayun, Sibtain Ahmed, Aysha Habib Khan

**Affiliations:** a Department of Pathology and Laboratory Medicine, Aga Khan University, Stadium Road, P.O. Box 3500, Karachi, 74800, Pakistan; b Department of Pediatrics and Child Health, Aga Khan University, Stadium Road, P.O. Box 3500, Karachi, 74800, Pakistan

**Keywords:** Thyroid stimulating hormone, Reference interval, Pakistan

## Abstract

**Introduction:**

Reference intervals (RIs) of thyroid-stimulating hormone (TSH) and free thyroxine (FT4) are age, assay and population specific. Currently, the age and assay-specific RIs for TSH are not available for children under two years of age. This study aimed to establish reference intervals for serum concentrations of TSH and FT4 in healthy children aged 1–24 months as per CLSI C28-A3 guidelines.

**Methods:**

This prospective cross-sectional study was conducted in children from 1 to 24 months visiting the clinical laboratory for serum vitamin D testing but without any recent illness, hospitalization, medication and history of maternal thyroid diseases from August 2018 to March 2019 were invited to participate in the study.

Serum TSH and FT4 were measured on ADVIA Centaur (Siemens Diagnostics, US), using chemiluminescence immunoassay. Kolmogorov–Smirnov test assessed normality of the data and RIs based on central 95% of the population were established using the non-parametric approach.

**Results:**

After excluding one subject with confirmed congenital hypothyroidism, a total of 131 children were included in the study. The median (IQR) age of the study subjects was 12 months (11), and majority 78 (59.5%) were boys. The RIs were established using non-parametric approach as the data was not normally distributed. Reference interval for TSH was 0.73–4.94 μIU/mL and for FT4 was 0.81–1.51 ng/dl.

**Conclusion:**

We established assay-specific RIs for serum TSH and FT4 in children aged 1–24 months in our population. The RIs were slightly lower from RIs developed on other platforms in different population.

## Highlights

• Reference intervals (RIs) of TSH and FT4 are age, assay and population specific.
• RIs of TSH & FT4 for <2 yrs Pakistani children on Advia Centaur analyzer not available.
• The RIs for TSH (0.73–4.94μIU/mL) and FT4 (0.81–1.51 ng/dl) for < 2 yrs old children.

## 1 Introduction

Congenital hypothyroidism (CH) occurs in approximately 1: 2,000 to 1: 4,000 newborns worldwide [1]. However, Pakistan's estimated incidence is much higher, approximately 1 in 1600 live births [2]. The CH is one of the most common preventable causes of mental retardation [3]. With early detection through newborn screening (NBS) and prompt treatment with levothyroxine, intellectual disability can be prevented [4].

Screening for CH is performed by dried blood spot screening of Thyroid Stimulating Hormone (TSH) within 48–72 h of birth. A positive screening test is confirmed by measuring serum TSH and free thyroxine (FT4) levels [5]. For a better intellectual outcome of patients with CH, thyroid hormone concentrations should be normalized rapidly (within the first two weeks after treatment initiation) and then maintained [6]. According to the American Academy of Pediatrics Guidelines, for optimal thyroxin replacement, serum TSH and FT4 measurements should be performed at regular intervals. More frequent monitoring is recommended when compliance is questioned, abnormal values are obtained, or dose or medication source has been changed [7].

Appropriate age, population and assay-specific TSH reference intervals (RIs) are essential for optimal treatment outcomes. The RIs of TSH are wider in neonates as compared to infants and other pediatric age groups, so if one uses the same neonatal RIs to interpret the results of children older than one month and vice versa, it will lead to under or over the treatment of the patient, leading to adverse effects and unnecessary repeated testing.

Guidelines by the National Academy of Clinical Biochemistry recommend that each laboratory determine their age and assay-specific TSH-RIs to ensure appropriate interpretation [8]. There was a need to establish RIs for our population in children under two years to improve clinical care. This study was conducted to establish RIs for serum TSH in healthy children under two years of age, as per the Clinical Laboratory Standards Institute (CLSI), C28-A3 guidelines. We also compare established cutoffs with reported reference data [9].

## 2 Material and methods

**Fig. 1:**
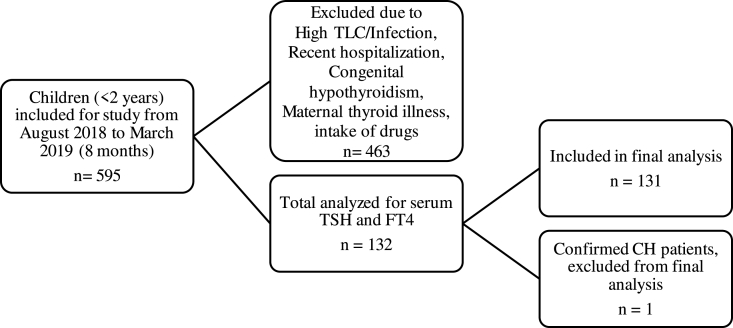
Flowchart of subjects included in the study.

A prospective cross-sectional study was conducted at the Section of Chemical Pathology, Department of Pathology and Laboratory Medicine, AKUH Karachi Pakistan after approval from Aga Khan University Hospital's Ethics Review Committee. The study has been reported in line with the STROCSS criteria [10]. This study is registered on ‘Clinicaltrials.gov’ and the unique Identifying number is NCT04877665. Healthy children from 1 to 24 months visiting the clinical lab for serum vitamin D testing from August 2018 to March 2019 were invited to participate in the study. Their health status was determined by asking questions related to recent illness, hospitalization, medication and maternal history of thyroid-related diseases (Appendix 1), after informed consent from their parents/guardian. Children with recent abnormal total leucocyte or neutrophil count in the medical record or positive microbial cultures, history of any diagnosed disease or infection, history of hospitalization during the previous four weeks, congenital hypothyroidism or transient congenital hypothyroidism, history of maternal thyroid illness, or any medications with potential influences on the thyroid function, such as amiodarone, anti-epileptic drugs, glucocorticoids were excluded, shown in Fig. 1.

### 2.1 Biochemical analysis

Residual serum samples received for serum vitamin D testing were utilized to measure serum TSH and FT4. Serum TSH and FT4 were analyzed on ADVIA® Centaur™, Siemens Diagnostics USA using chemiluminescence immunoassay technique. The ADVIA Centaur TSH-Ultra (3rd generation) assay's analytical measurement range was from 0.010 to 150 μIU/mL (1uIU/mL = 1mIU/L), while for FT4 was 0.1–12.0 ng/dl. The TSH assay standardization was traceable to the World Health Organization (WHO) 3rd International Standard for human TSH (IRP 81/565), so results were not procedure dependent. Three-level quality control materials were run with each batch of samples for internal quality control. External proficiency was assured by analyzing samples from the College of American Pathologists thrice per year, throughout the study period, and all external proficiency surveys during the study period were acceptable. The manufacturer provided TSH RIs were for >2 years to adult age-group only, while the manufacturer provided RI for FT4 for children of 1–23 months was 0.94–1.44 ng/dl.

The RIs for children aged 1–24 months were established using the CLSI, C28-A3 guidelines 'Defining, Establishing, and Verifying Reference Intervals in the Clinical Laboratory; Approved Guideline Third Edition'. These guidelines recommend using a sample size of at least 120 specimens, to obtain both an RI estimate and a 90% confidence interval for it without making any assumption about the population distribution. The sample size was kept at 132, after adding an attrition rate of 14%. Ethical approval was sought and obtained from the institution's Ethical Review Committee before the study's commencement (ERC number: 5255-Pat-ERC-18).

### 2.2 Statistical analysis

The EP evaluator version 10 and SPSS version 21 were used for data analysis. Kolmogorov–Smirnov test assessed the normality of the data. The RIs based on central 95% of the population were established using the nonparametric approach. In the nonparametric method, the reference interval is calculated, making no assumption about the population distribution shape. The median age of subjects, TSH and FT4 levels were calculated. For assessing differences in the male and female gender, Students' t' test was applied, taking p-value <0.050 as significant. Spearman's test computed the correlation between age, TSH and FT4 levels.

## 3 Results

A total of 132 children were included in the study; one subject with confirmed congenital hypothyroidism (CH) was excluded from the study, shown in Fig. 1. The TSH and FT4 values of 131 subjects were included to establish the RIs. The median (IQR) age of the study subjects was 12 (11) months, and 78 (59.5%) were male. The median (95% intervals) of serum TSH and free T4 were 1.94 (0.61–6.91) μIU/L and 1.09 (0.68–1.73) ng/dL respectively.

The data was not normally distributed, so the non-parametric approach was applied to establish RIs. Reference interval based on central 95% of the population for TSH is 0.73–4.94 μIU/mL and for FT4 is 0.81–1.51 ng/dL. The RIs for Male and female were not significantly different for both TSH (p-value 0.096) and FT4 (p-value 0.878), (Table 1).

**Table 1:** Reference interval for thyroid stimulating hormone and free T4 in children under 2 Years of age (n = 131).

		Range	Median	P-value	Lower Limit (95% CI)	Upper Limit (95% CI)
**TSH (μIU/mL)**	**Male (n = 78)**	0.61–4.95	1.87	0.096	0.67 (0.55–0.81)	4.31 (3.81–4.86)
	**Female (n = 53**	0.86–6.91	2.13		0.75 (0.61–0.92)	5.75 4.70–7.03
	**Both (n = 131)**	0.61–6.91	1.94		**0.73 (**0.61–0.86)	**4.94** 4.41–6.91
**FT4 (ng/dl)**	**Male (n = 78)**	0.68–1.73	1.09	0.878	0.80 (0.76–0.84)	1.47 1.40–1.54
	**Female (n = 53)**	0.75–1.53	1.09		0.77 (0.70–0.83)	1.43 1.37–1.50
	**Both (n = 131)**	0.68–1.73	1.09		**0.81 (**0.68–0.84)	**1.51** 1.42–1.73

Both TSH (p value 0.000, r = −0.339) and FT4 (p value 0.006, r = −0.239) levels significantly correlated with age, the levels decreasing with increasing age, Fig. 2(a), Fig. 2 (b)(a) and (b).

**Fig. 2(a):**
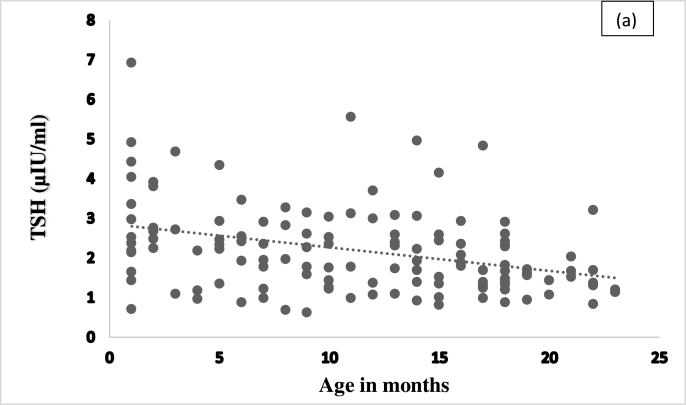
Age-wise distribution of Thyroid Stimulating Hormone levels in study subjects.

**Fig. 2 (b):**
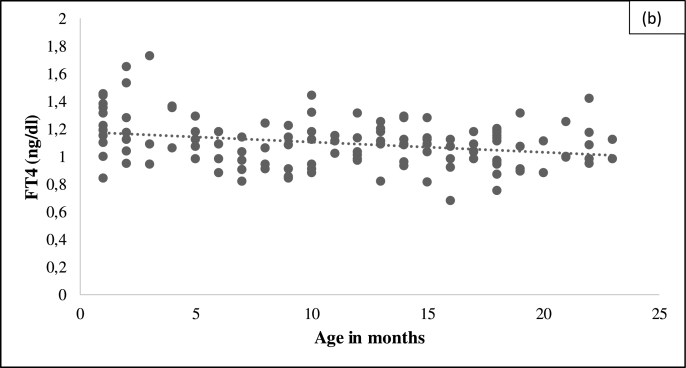
Age-wise distribution of Free T4 levels in study subjects.

Subjects were divided into three age groups, Group I; <6 months, group II; 7–12 months and group III; 13–24 months. The mean difference between TSH and FT4 levels between these age groups was statistically significantly (p-value 0.001 and p-value <0.001) respectively (Table 2).

**Table 2:** Distribution of Thyroid Stimulating Hormone and Free T4 in different age groups (n = 131).

-	<6 months	7–12 months	13–24 months	p value
**n**	37	34	60	–
**TSH (uIU/ml)**	2.72 ± 1.3	2.12 ± 1.06	1.88 ± 0.88	0.001
**FT4 (ng/dl)**	1.19 ± 0.2	1.05 ± 0.14	1.06 ± 0.14	<0.001

## 4 Discussion

CH is one of the most common preventable causes of mental retardation and satisfies all the criteria for being included in an NBS program [11]. After a CH diagnosis, all children should be treated and frequently monitored using serum TSH and FT4. For accurate monitoring, appropriate RIs must be used for reporting the patient's results. The RIs of TSH is affected by age, gender, population, iodine intake and assay used [8].

This study was conducted to establish assay and population-specific RIs for children aged 1–24 months, as RIs for this age group were not available. The RIs determined by the present study for TSH and FT4 were 0.73–4.94 μIU/mL and 0.81–1.51 ng/dl, respectively. No studies have reported assay and population-specific RIs for the Pakistani, Indian or even South Asian population to the best of our knowledge. However, the clinical practice guidelines for screening, diagnosis and management of CH by the Indian Society for Pediatric and Adolescent Endocrinology has recommended the RIs determined by Lem AJ et al. in a European population of Netherlands [6,12]. The RIs determined by Lem AJ et al. for 1–3 months, 3–12 months, 1–5 years for TSH were 0.58–5.57 mIU/L, 0.57–5.54 mIU/L, 0.56–5.41 mIU/L and FT4 were 1.04–3.4 ng/dl, 1.1–2.4 ng/dl, 1.1–2 ng/dl respectively. These RIs are slightly wider than the RIs established in our population, the difference could have been due to a different assaying platform utilized, i.e. Advia Centaur immunoassay analyzer (Siemens Diagnostics) used in current study while Vitros ECI immunoassay analyzer (Ortho Diagnostics) used by Lem AJ et al. When comparing with RIs developed on a similar platform as used in the present study, i.e. Advia Centaur immunoassay analyzer (Siemens Diagnostics), but different population we observed that the RIs for our population were slightly lower as compared to Caucasians and Asians shown in Table 3 [[13], [14], [15]]. Loh TP et al. established TSH RIs in Asian children, including 59% Chinese and 13% Indians using Advia Centaur immunoassay in 82% of the subjects [15].

**Table 3:** The RIs determined by Kapelri et al. (12), Hubner et al. (13) and Loh et al. (14) using Advia Centaur Immunoassay analyzer.

Study	Our Study	Kapelari et al.		Hubner et al.		Tze Ping Loh et al.
Country	Pakistan	Austria		Germany		Singapore
Analyzer	Advia Centaur	Advia Centaur		Advia Centaur		Advia Centaur and Ortho Vitros 5600
**Age**	1–24 Months	1m–12 m	1y-2y	1m-1y	1y-5y	2m-4y
**FT4 (ng/dl)**	0.79–1.34	0.92–1.74	0.92–1.61	0.88–1.62	0.88–1.47	–
**TSH (μIU/ml)**	0.733 4.945	1.30–7.09	1.0–5.42	0.30–5.88	1.0–5.42	0.74–5.68

The trends of the reference values obtained from this study showed slight differences from those obtained from the other studies, which might be explained by different analytical assays, different tools used for statistical analysis, differences in ethnicity, population or geographic derived covariates such as lifestyle, salt iodination, and nutrition. These findings further endorse that there was a dire need to develop assay-specific RIs for our population. The RIs established in subjects from the same geographic area, using the same analyzer used in routine work, is definitively better.

We observed no significant difference in TSH or FT4 RIs between male and female in the present study. However, RIs significantly correlated with age, i.e. decreasing RIs with increasing age, Table 2. Similar findings were reported by Kratzsch J. et al. which assessed the association between TSH, age, BMI, Number of blood leucocytes, gender and observed that only age was a relevant predictor of TSH variation with 8% [16]. Therefore, these findings suggest that the establishment of age-dependent reference intervals is mandatory for TSH and larger, prospective studies should be performed to address these findings.

This is the first study from Pakistan reporting the RIs for TSH and FT4 for children aged 1–24 months using the Advia® Centaur™ assay. One of the strengths of this study was the prospective study design. Collecting pediatric healthy subjects samples is especially difficult, and most of the studies reported in the literature have established TSH and FT4 RIs for pediatric age groups using retrospective data analysis. Our study presents some limitations, including small sample size, non-partitioning of the data into smaller age groups, and due to limited availability of funds thyroid, antibody testing of mother and child could not be performed. A more extensive prospective study needs to be planned with further age categorization for a more generalizable data.

## 5 Conclusion

We established assay-specific RIs for serum TSH and FT4 in children aged 1–24 months in our population. The RIs developed differed slightly from RIs developed using other platforms in different population. The TSH and FT4 were significantly associated with age, requiring that validated RIs developed for our population should be used for assessing TSH and FT4 results in children under two years of age. However same RIs of TSH and FT4 can be used for both male and female children.

## Availability of data and materials

The data set used and analyzed in the current study are available from the corresponding author upon reasonable request.

## Ethical Approval

ERC Number: 5255-Pat-ERC-18.

## Sources of funding

This project was funded by Departmental Resident Research Grant 2018 by 10.13039/100015537Department of Pathology and Laboratory Medicine, Aga Khan University, Karachi Pakistan.

## Author contribution

SM & HM: Conception, design of study, data collection, literature review and manuscript writing.

IS, AHK, LJ, & KNH: Drafting the work and revising it critically for important intellectual content.

All authors approved the final manuscript.

## Research registration number

Name of the registry: Clinicaltrials.gov

Unique Identifying number or registration ID: NCT04877665

Hyperlink to your specific registration (must be publicly accessible and will be checked): https://register.clinicaltrials.gov/prs/app/action/SelectProtocol?sid=S000AYB9&selectaction=Edit&uid=U0005NQI&ts=2&cx=kgj9de

## Guarantor

Dr. Hafsa Majid.

Provenance and peer review

Not commissioned, externally peer-reviewed

## Declaration of competing interest

The authors declare that they have no competing interests.
