# Supplementary material for: Establishing reference interval for thyroid-stimulating hormone in children below two-year ages in Pakistani population
Source: Ann Med Surg (Lond). 2021 Jul 31;68:102601. doi: 10.1016/j.amsu.2021.102601 (PMC8350183; doi:10.1016/j.amsu.2021.102601)
Supplement: Multimedia component 1 [file mmc1.docx]

**Appendix 1: Questionnaire to Assess the Health Status of Children. (Supplemental material)**

| S# | Question | √ if Present |
| --- | --- | --- |
| 1 | Is your child healthy?  کیا آپکا بچہ صحت مند ہے؟ |  |
| 2 | Has your child been ill in past 2 weeks?  کیا آپ کا بچہ گزشتہ دو ہفتوں میں بیمار ہوا ہے؟ |  |
| 3 | Was your child hospitalized due to serious illness in past 2 month?  کیا آپکا بچہ گزشتہ 2 مہینے میں سنگین بیماری کی وجہ سے ہسپتال گیا تھا؟ |  |
| 4 | Does the mother have any thyroidal illness?  کیا ماں کوکوئی ladioryht بیماری ہے؟  If yes, Was she on any medication during her pregnancy?  اگر ہاں، کیا ماں حمل کے دوران کسی بھی دوا پر تھی؟  If Yes, Name of Medicine:  اگر ہاں، دوا کا نام |  |
| 5 | Does any other family member have any thyroidal illness?  کیا خاندان کے کسی رکن کوکوئی thyroidal بیماری ہے؟  If Yes, type of thyroid disease:  اگر ہاں، بیماری کی قسم: |  |
| 6 | When was the last meal taken?  بچے نےآخری کھانا کب لیا تھا؟ |  |
| 7 | Is your child taking any medication?  کیا آپ کا بچہ کوئی دوا لے رہا ہے؟ |  |
| 8 | Is your child on any supplement?  کیا آپ کا بچہ کسی بھی tnemelppus پر ہے؟  Supplement & Dose: ____________________  سپلیمنٹ اور دوا کی خوراک  Duration:  دورانیہ: |  |
| 9 | Serum TSH performed at birth?  سیرم TSH پیدائش میں انجام دیا ہے؟  If Yes, TSH levels:  TSH کی سطح: |  |
